# Supplementary material for: Molecular Mechanisms of Succinimide Formation from Aspartic Acid Residues Catalyzed by Two Water Molecules in the Aqueous Phase
Source: Int J Mol Sci. 2021 Jan 6;22(2):509. doi: 10.3390/ijms22020509 (PMC7825500; doi:10.3390/ijms22020509)
Supplement: Supplementary file 1 [file ijms-22-00509-s001.pdf]

Supplemental Materials: Molecular Mechanisms of Succinimide Formation from Aspartic Acid Residues Catalyzed by Two Water Molecules in the Aqueous Phase

Tomoki Nakayoshi [1,2], Koichi Kato [1,3], Shuichi Fukuyoshi [2], Ohgi Takahashi [4], Eiji Kurimoto [1] and Akifumi Oda [1,2,5,*]

[1] Graduate School of Pharmacy, Meijo University, 150 Yagotoyama, Tempaku-ku, Nagoya, Aichi 468-8503, Japan; 184331503@ccmailg.meijo-u.ac.jp (T.N.); kato-k@kinjo-u.ac.jp (K.K.); kurimoto@meijo-u.ac.jp (E.K.)

[2] Institute of Medical, Pharmaceutical and Health Sciences, Kanazawa University, Kakuma-machi, Kanazawa, Ishikawa 920-1192, Japan; fukuyosi@p.kanazawa-u.ac.jp

[3] Department of Pharmacy, Kinjo Gakuin University, 2-1723 Omori, Moriyama-ku, Nagoya, Aichi 463-8521, Japan

[4] Faculty of Pharmaceutical Sciences, Tohoku Medical and Pharmaceutical University, 4-4-1 Komatsushima, Aoba-ku, Sendai, Miyagi 981-8558, Japan; ohgi@tohoku-mpu.ac.jp

[5] Institute for Protein Research, Osaka University, 3-2 Yamadaoka, Suita, Osaka 565-0871, Japan

* Correspondence: oda@meijo-u.ac.jp; Tel.: +81-52-832-1151

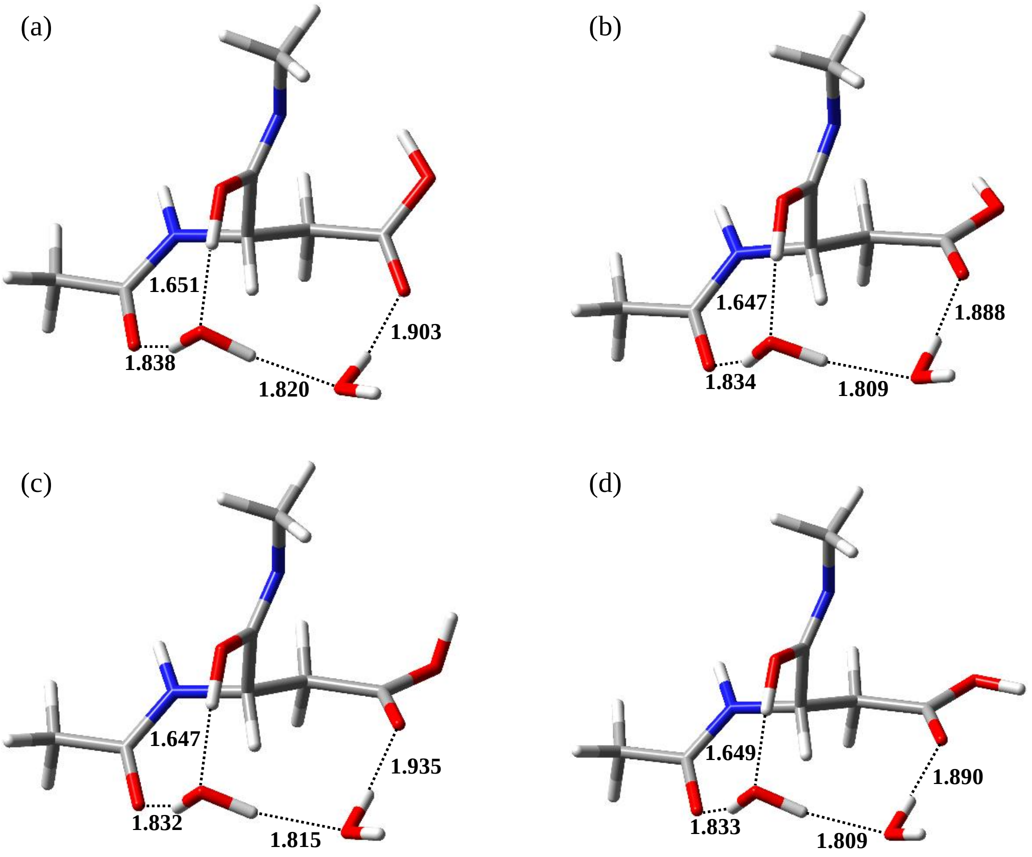

Figure S1. Optimized geometries of (**a**) TS3 (φ = −113°, ψ = −96°, χ_1 = −176°), (**b**) IM2 (φ = −114°, ψ = −97°, χ_1 = −172°), (**c**) TS4 (φ = −114°, ψ = −98°, χ_1 = −171°), and (**d**) IM3 (φ = −113°, ψ = −97°, χ_1 = −172°). Selected interatomic distances are presented in Å. Carbon, hydrogen, nitrogen, and oxygen atoms are illustrated in gray, white, blue, and red, respectively.

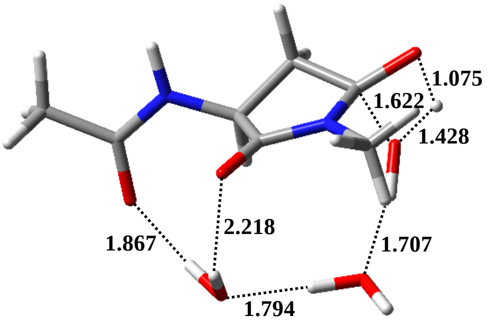

Figure S2. Optimized geometries of TS of the direct pathway for the formation of the Suc residue from TH1 (φ = −97°, ψ = −149°, χ_1 = 158°). Selected interatomic distances are presented in Å. Carbon, hydrogen, nitrogen, and oxygen atoms are illustrated in gray, white, blue, and red, respectively.
